# Antiangiogenic exclusion rules in glioma trials: Historical perspectives and guidance for future trial design

**DOI:** 10.1093/noajnl/vdae039

**Published:** 2024-03-15

**Authors:** Ugur Sener, Mahnoor Islam, Mason Webb, Sani H Kizilbash

**Affiliations:** Department of Neurology, Mayo Clinic, Rochester, Minnesota, USA; Department of Neurology, Medical University of South Carolina, Charleston, South Carolina, USA; Department of Medical Oncology, Mayo Clinic, Rochester, Minnesota, USA; Department of Medical Oncology, Mayo Clinic, Rochester, Minnesota, USA

**Keywords:** antiangiogenic, bevacizumab, clinical trial, glioma, study design

## Abstract

**Background:**

Despite the lack of proven therapies for recurrent high-grade glioma (HGG), only 8%–11% of patients with glioblastoma participate in clinical trials, partly due to stringent eligibility criteria. Prior bevacizumab treatment is a frequent exclusion criterion, due to difficulty with response assessment and concerns for rebound edema following antiangiogenic discontinuation. There are no standardized trial eligibility rules related to prior antiangiogenic use.

**Methods:**

We reviewed ClinicalTrials.gov listings for glioma studies starting between May 2009 and July 2022 for eligibility rules related to antiangiogenics. We also reviewed the literature pertaining to bevacizumab withdrawal.

**Results:**

Two hundred and ninety-seven studies for patients with recurrent glioma were reviewed. Most were phase 1 (*n* = 145, 49%), non-randomized (*n* = 257, 87%), evaluated a drug-only intervention (*n* = 223, 75%), and had a safety and tolerability primary objective (*n* = 181, 61%). Fifty-one (17%) excluded participants who received any antiangiogenic, one (0.3%) excluded participants who received any non-temozolomide systemic therapy. Fifty-nine (20%) outlined washout rules for bevacizumab (range 2–24 weeks, 4-week washout *n* = 35, 12% most common). Seventy-eight required a systemic therapy washout (range 1–6 weeks, 4-week washout *n* = 34, 11% most common). Nine permitted prior bevacizumab use with limitations, 18 (6%) permitted any prior bevacizumab, 5 (2%) were for bevacizumab-refractory disease, and 76 (26%) had no rules regarding antiangiogenic use. A literature review is then presented to define standardized eligibility criteria with a 6-week washout period proposed for future trial design.

**Conclusions:**

Interventional clinical trials for patients with HGG have substantial heterogeneity regarding eligibility criteria pertaining to bevacizumab use, demonstrating a need for standardizing clinical trial design.

**Key Points**

- Antiangiogenic-based eligibility rules used in glioma clinical trials are inconsistent.
- Bevacizumab discontinuation for disease progression can result in rebound edema, but this is not universal.
- Six-week washout is likely to be tolerated, permitting the timely introduction of experimental therapeutics.

**Importance of the Study**

Given limited treatment options for patients with recurrent glioma, every patient should be considered for clinical trial enrollment. Excessively strict exclusion criteria can act as barriers to participation. Prior treatment with bevacizumab is frequently cited as an exclusion criterion in clinical trials for patients with recurrent glioma. In this study, we reviewed ClinicalTrials.gov listings for glioma studies starting between May 2009 and July 2022 for eligibility rules related to antiangiogenics. We found that antiangiogenic-based eligibility rules are inconsistent and heterogeneous. We also reviewed the literature pertaining to the clinical impact of bevacizumab withdrawal in multiple cancer scenarios in context with bevacizumab pharmacokinetics and pharmacodynamics. We found that a 6-week washout is likely to be tolerated by most patients, permitting timely introduction of experimental therapeutics. These findings can inform future clinical trial design, allow the use of more permissive inclusion criteria that can improve participation and expedite development of novel therapeutics.

Despite years of research in basic science and clinical trials, treatment of patients with adult-type gliomas remains a clinical challenge. Following initial multimodality therapy involving surgical resection, radiation therapy, and systemic therapy, tumors invariably recur and represent life-limiting illnesses.^1–3^ Treatment of recurrent glioblastoma (GBM) or other high-grade glioma (HGG) is not standardized with no single agent to date conferring a clear survival benefit.^2^ In the absence of viable alternatives readily available in the clinic, many patients with recurrent HGG are treated with antiangiogenics. Bevacizumab is an anti-vascular endothelial growth factor (VEGF) antibody that is US Food and Drug Administration (FDA) approved for adult patients with progressive GBM.^4^ However, due to the lack of survival benefits associated with the agent, the European Medicines Agency has rejected this indication.^5^ In a pooled analysis of patients with newly diagnosed GBM, bevacizumab was associated with improved progression-free survival (PFS), but not overall survival (OS).^6^ In the setting of recurrent GBM, bevacizumab alone or in combination with lomustine or irinotecan was associated with radiographic responses and improved PFS, but use of the antiangiogenic agent did not improve OS.^7,8^ Despite lack of established survival benefits for GBM or other HGG, bevacizumab is frequently utilized as a steroid-sparing strategy to manage peritumoral edema and to manage radiation necrosis.^7–10^

Given limited treatment options for patients with recurrent HGG, every patient with this diagnosis should be considered for clinical trial enrollment.^11^ This is especially the case at first recurrence, as these patients are most likely to have a good performance status and thus sustain benefit from novel therapies. Yet only an estimated 8%–11% of patients with GBM enroll in clinical trials.^12^ While estimates are not available, participation is likely to be lower for other HGGs due to smaller number of studies available. Although poor performance status, cognitive issues, and other patient variables are associated with the low participation of HGG patients in clinical trials, excessively strict exclusion criteria may also act as barriers to patient participation. As an example, previous participation in a clinical trial for newly diagnosed tumors may preclude individuals from participating in studies for recurrent disease. Prior treatment with bevacizumab is also frequently cited as an exclusion criterion in clinical trials for patients with recurrent glioma.^2^ Recurrent cerebral edema following cessation of bevacizumab has been reported, which can result in clinical decline and premature termination of study participation.^13,14^ Additionally, the increase in contrast enhancement or T2 hyperintensity on MRI following bevacizumab discontinuation may indicate rebound edema rather than tumor progression, thus confounding response assessments.^13,15^ Although these are valid concerns, the methodology of clinical trial exclusion for current or prior antiangiogenic therapy is not standardized. Many clinical trials either exclude patients with prior antiangiogenic use altogether or require arbitrarily defined extended washout periods prior to enrollment. Beyond prolonging trial duration due to slower accrual, other consequences of such eligibility criteria include patients and caregivers declining antiangiogenic therapy to maintain eligibility for potential future studies. This may negatively impact patient care and outcomes. A standardized scientifically rational approach to handling prior antiangiogenic therapy is thus critical for optimal patient care and trial enrollment and will expedite the development of therapeutics for patients with glioma. In this study, we first sought to review the heterogeneity of eligibility patterns pertaining to prior antiangiogenic use in clinical trials involving patients with recurrent glioma. Thereafter we systematically reviewed the literature pertaining to the clinical impact of bevacizumab withdrawal, with the goal of providing guidance to clinical trialists on future study design, pertaining to eligibility criteria for prior bevacizumab use.

## Materials and Methods

### Study Selection

We searched ClinicalTrials.gov listings for phase 1, 2, and 3 trials involving adult patients with glioma with study starts between May 5, 2009 and July, 15 2022. May 5, 2009 is the date bevacizumab was granted accelerated approval by the FDA for patients with recurrent glioblastoma^.4^ The specific search criteria used are defined in Table 1. Over the search period, World Health Organization (WHO) classification of CNS tumors was updated in 2016 and 2021.^16,17^ For the purposes of this analysis, trials for all adult-type diffuse gliomas under the current CNS WHO classification including astrocytoma, IDH-mutant; oligodendroglioma, IDH-mutant, and 1p/19q-codeleted; and glioblastoma, IDH-wild type were considered.^17^ Separate searches were conducted for astrocytoma and oligodendroglioma to ensure inclusion of relevant studies for these specific tumor types over the search period.

**Table 1.** ClinicalTrials.gov Study Inclusion Criteria

Criterion	Value^*^
Condition or disease	Glioblastoma Glioma Recurrent glioblastoma Recurrent glioma
Status	Not yet recruiting Recruiting Enrolling by invitation Active, not recruiting Completed
Age	Adult (18–64) Older adult (65+)
Phase	Phase 1 Phase 2 Phase 3
Study start	From May 5, 2009 To July 15, 2022

^*^All search values per criterion conducted using the “OR” search operator.

**Figure 1.**
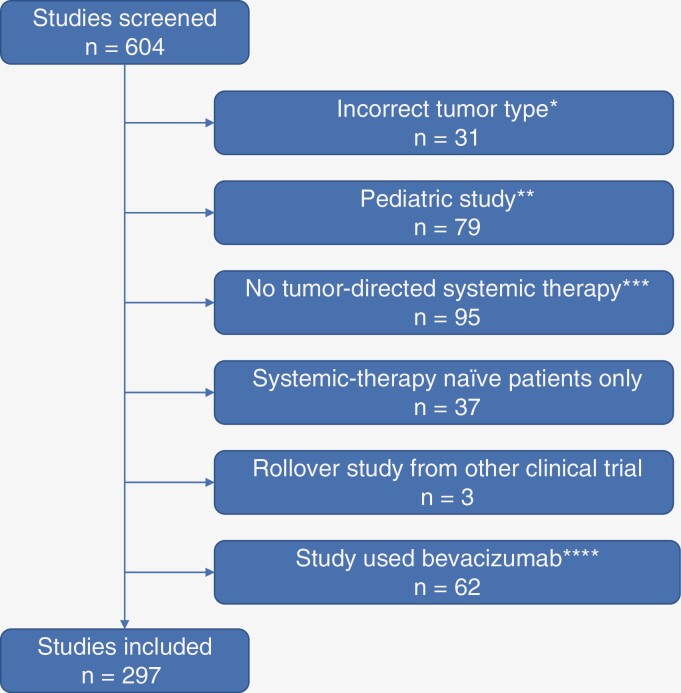
Study selection. *Studied tumors other than astrocytoma, oligodendroglioma, or glioblastoma. **Studies targeting patients up to age 21. ***Studies investigating imaging techniques, supportive care measures, surgical interventions, radiation therapy interventions, and caregiver measures. ****Studies using bevacizumab, either as a treatment or control arm.

Six hundred and four resultant studies were screened independently by 2 authors (U.S. and M.I.) with data validated by a third author (M.W.; Figure 1). Thirty-one studies for patients with diagnoses other than glioblastoma, astrocytoma, and oligodendroglioma were excluded. These studies were for medulloblastoma (*n* = 15), ependymoma (*n* = 10), neurofibromatosis type 2 associated tumors (*n* = 3), diffuse midline glioma H3K27-altered (*n* = 1), neurofibromatosis type 1 associated neurofibromas (*n* = 1), neuroendocrine tumors (*n* = 1), rhabdoid/teratoid tumor (*n* = 1) and subependymal giant cell astrocytoma (*n* = 1), with 2 studies including more than one of the listed tumor types. Seventy-nine studies designed for pediatric patients up to age 21 were excluded. Ninety-five studies that did not investigate a tumor-directed pharmacological agent for recurrent glioma were excluded. These studies investigated imaging techniques (*n* = 45), supportive care measures (*n* = 24), surgical interventions (*n* = 17), radiation therapy interventions (*n* = 6), and caregiver measures (*n* = 3).

Prior use of bevacizumab was not applicable to 37 studies designed for patients naïve to any form of systemic therapy (eg, newly diagnosed glioma) or 3 studies that involved patients rolling over from a different clinical trial to continue experimental systemic therapy. Sixty-two studies that used bevacizumab either as a treatment or comparison arm were also excluded. The remaining 297 studies were reviewed for patient eligibility rules related to prior bevacizumab use.

### Study Review

Each study selected for inclusion was reviewed independently by 2 authors (U.S. and M.I.) with resultant data validated by a third author (M.W.). Data collected included study National Clinical Trial (NCT) number, study URL, title, start date, completion date, study phases, treated conditions, type of intervention, intended enrollment, funding source, randomization status, and primary outcome measures.

Trial inclusion and exclusion criteria were reviewed to identify rules related to prior bevacizumab use. Studies excluding participants who received bevacizumab, or any other antiangiogenic therapy were noted. Remaining studies were reviewed for rules related to washout from specific bevacizumab or other systemic therapy. Studies where no washout period was specified were noted.

No local Institutional Review Board approval was sought for this study analyzing publically available clinical trial data as posted on ClinicalTrials.gov.

## Results

Two-hundred-ninety-seven studies where a therapeutic intervention was undertaken for patients with recurrent glioblastoma, astrocytoma, or oligodendroglioma were reviewed for eligibility criteria related to prior antiangiogenic use. Findings are summarized in Table 2.

**Table 2.** Summary of Recurrent Glioma Clinical Trials

Characteristic	*n* (%)
Total studies reviewed	297
Conditions treated	
HGG only	163 (55%)
HGG and other gliomas	45 (15%)
HGG and other CNS tumors	28 (9%)
HGG and other systemic tumors	61 (21%)
Phase	
Early phase 1^*^	24 (8%)
Phase 1	145 (49%)
Phase 1/2	44 (15%)
Phase 2	76 (26%)
Phase 3	3 (1%)
Not applicable^**^	5 (2%)
Anticipated enrollment	
Less than 11	33 (11%)
11–20 21–30 31–40 41–50 51–100	56 (19%) 49 (16%) 41 (14%) 27 (9%) 48 (16%)
101–200	28 (9%)
Greater than 201	15 (5%)
Randomization	
Randomized	40 (13%)
Non-randomized	257 (87%)
Intervention	
Drug only	223 (75%)
Drug and procedure	3 (1%)
Drug and radiation	9 (3%)
Device only	3 (1%)
Device and drug	6 (2%)
Vaccine	22 (7%)
Viral vector	16 (5%)
T cells	14 (5%)
NK cells	1 (0.3%)
Primary objective	
PK, PD, tissue concentration based^***^	42 (14%)
Safety and tolerability	181 (61%)
Response rate	36 (12%)
Progression-free survival	30 (10%)
Overall survival	8 (3%)
Study start date	
May 5, 2009—December 31, 2014	99 (33%)
January 1, 2015—December 31, 2019	111 (37%)
January 1, 2019—July 15, 2022	87 (29%)
Study results	
No results available	251 (85%)
Has results	46 (15%)

HGG, high-grade glioma; CNS, central nervous system; NK cell, natural killer cell; PK, pharmacokinetics; P, pharmacodynamics; TMZ, temozolomide.

^*^According to the National Institutes of Health, early phase 1 refers to exploratory trials conducted before traditional phase 1 trials. Formerly, these trials were listed as phase 0.

^**^On ClinicalTrials.gov, “Not Applicable” is used to describe trials without FDA-defined phases. This would typically include trials with a device or behavioral intervention.

^***^PK, PD, tissue concentration based describes studies where the stated primary objective was to gain information about drug pharmacokinetics, pharmacodynamics, metabolism, and tissue uptake.

Of 297 studies reviewed, 163 (55%) were for patients with HGG only. The remaining studies were open to patients with HGG in addition to other gliomas (*n* = 45, 15%), any CNS tumor (*n* = 28, 9%), or solid organ tumors including HGG (*n* = 61, 21%). Most of the studies were phase 1 only (*n* = 145, 49%), followed by phase 2 only (*n* = 76, 26%), phase 1/2 (*n* = 44, 15%), and early phase 1 (*n* = 24, 8%). Only 40 trials (13%) were randomized. Safety and tolerability were the stated primary objectives of most studies (*n* = 181, 61%) consistent with the large number of phase 1 clinical trials included. Forty-two (14%) of the studies had a pharmacokinetics, pharmacodynamics, or tissue concentration-based primary objective. Other primary objectives included response rate (*n* = 36, 12%), PFS (*n* = 30, 10%), and overall survival (OS; *n* = 8, 3%).

Most studies evaluated a drug-only intervention (*n* = 223, 75%). Other interventions included drugs and procedures such as laser interstitial thermal therapy (*n* = 3, 1%), drug and radiation (*n* = 9, 3%), medical devices such as tumor treating fields (*n* = 3, 1%), medical device and drug (*n* = 6, 2%), therapeutic vaccine (*n* = 22, 7%), viral vector (*n* = 16, 5%), T-cell therapy (*n* = 14, 5%), and NK cell therapy (*n* = 1, 0.3%). Most of the studies had no results available through the ClinicalTrials.gov website (*n* = 251, 85%). The majority of the studies were funded by industry only (*n* = 86, 29%) or a combination of industry and other sources such as National Institutes of Health (NIH) funding (*n* = 79, 27%; Supplementary Table 1).

A wide range of exclusion rules regarding prior antiangiogenic or other systemic therapy use were represented (Table 3). Thirty studies excluded participants who received any bevacizumab (*n* = 30, 10%) and 21 studies excluded participants who received any antiangiogenic therapy (*n* = 21, 7%). Fifty-nine (20%) studies outlined washout rules for patients who received bevacizumab prior to enrollment. The washout duration ranged from 2 weeks to 24 weeks with a 4-week washout (*n* = 35, 12%) being most common (Table 4). Seventy-eight studies did not have bevacizumab-specific rules but outlined washout rules for systemic therapy in general. The washout duration ranged from 1 to 6 weeks or 3 to 5 half-lives, with a 4-week washout (*n* = 34, 11%) being most common again. Nine studies permitted prior bevacizumab use with specific restrictions. Within this group, 7 studies allowed prior bevacizumab use only if the agent was administered for radiation necrosis or symptom management. One study permitted prior bevacizumab only if it was administered as part of first-line therapy and another permitted prior bevacizumab only if it was administered intra-arterially. Eighteen studies (6%) permitted any prior bevacizumab use whereas 5 (2%) studies were exclusively open to patients with bevacizumab-refractory disease. Seventy-six studies (26%) had no explicit rule about prior bevacizumab use.

**Table 3.** Clinical Trial Antiangiogenic Rules

Antiangiogenic rule	*n* (%)
Excludes participants who received any antiangiogenic	21 (7%)
Excludes participants who received any bevacizumab	30 (10%)
Excludes participants who received any non-TMZ systemic therapy	1 (0.3%)
Requires specific washout period for bevacizumab	59 (20%)
Requires washout period for prior systemic therapy	78 (26%)
Permit prior bevacizumab use with restrictions	9 (3%)
Permit any prior bevacizumab use	18 (6%)
Study for bevacizumab-refractory disease only	5 (2%)
No explicit rule regarding prior antiangiogenic use	76 (26%)

**Table 4.** Prior Therapy Washout Periods

Characteristic	*n* (%)
Total studies reviewed	297
Studies requiring specific washout period for bevacizumab	59 (20%)
2 weeks	3 (1%)
3 weeks	2 (0.6%)
4 weeks	35 (12%)
5 weeks	3 (1%)
6 weeks	6 (2%)
12 weeks	4 (1.3%)
14 weeks	1 (0.3%)
16 weeks	2 (0.6%)
24 weeks	3 (1%)
Studies requiring washout period for prior systemic therapy	78 (26%)
1 week	6 (2%)
2 weeks	12 (4%)
3 weeks	19 (6%)
4 weeks	34 (11%)
6 weeks	2 (0.6%)
3 half-lives (approximately 9 weeks for bevacizumab)	3 (1%)
4 half-lives (approximately 11 weeks for bevacizumab)	1 (0.3%)
5 half-lives (approximately 14 weeks for bevacizumab)	1 (0.3%)

**Figure 2.**
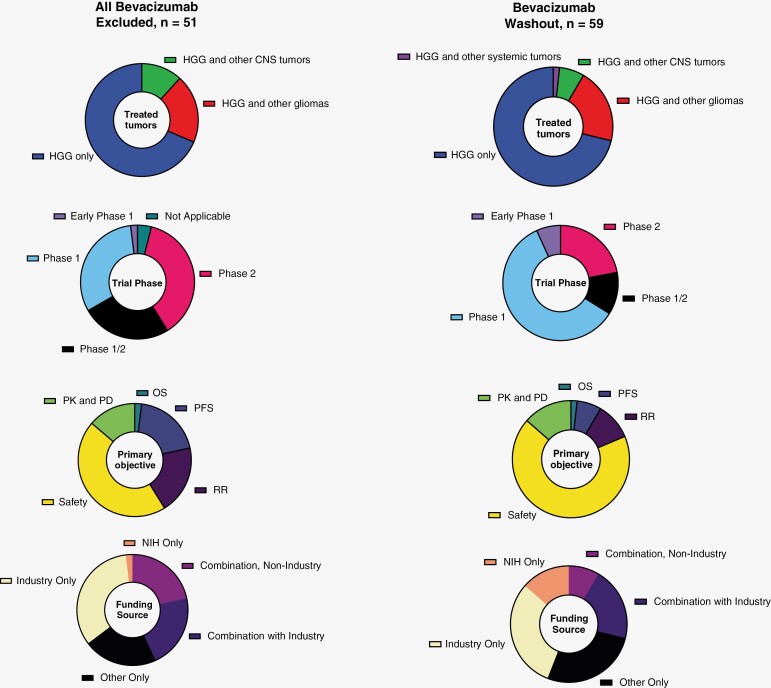
Analysis of studies that excluded any prior antiangiogenic use or mandated specific washout periods for participants previously receiving bevacizumab. HGG, high-grade glioma; CNS, central nervous system; PK, pharmacokinetics; PD, pharmacodynamics; OS, overall survival; PFS, progression-free survival; RR, response rate. On ClinicalTrials.gov, “Not Applicable” is used to describe trials without FDA-defined phases. This would typically include trials with a device or behavioral intervention. Other indicates studies funded by institutions other than industry and NIH. Combination, non-industry includes studies funded by NIH, other U.S. Federal Government funding, or other non-industry sources such as academic institutions.

Among the 51 studies that excluded patients who had any prior bevacizumab or other antiangiogenic therapy, 1 (2%) was early phase 1, 16 (31%) were phase 1, 13 (25%) were phase 1/2, and 19 (37%) were phase 2 (Figure 2). Thirty-five (69%) of these studies were for patients with HGG only, 10 (20%) were open to patients with other gliomas and 6 (12%) were open to patients with other CNS tumors. Forty-one (80%) had a drug-only intervention with the remainder studying other types of interventions. Fourteen (27%) had a start date between May 2009 and December 2014, within 5 years of bevacizumab FDA approval. Twenty-two (43%) had a start date between January 2015 and December 2019, with the remaining 15 (29%) starting in 2020 or later. Based on expected enrollment numbers at the time of our data search, these 51 studies anticipated the participation of 3234 patients who would have been ineligible in event of any bevacizumab or other antiangiogenic use.

Among the 59 studies that had specific washout rules regarding bevacizumab use, the most common washout period listed was 4 weeks (*n* = 35, 59%). Only 5 (8%) studies required a washout of less than 4 weeks in duration. Nineteen (32%) studies required a washout that was greater than 4 weeks. 4 (7%) were early phase 1, 35 (59%) were phase 1, 7 (12%) were phase 1/2, and 13 (22%) were phase 2 (Figure 2). Forty-two (71%) of these studies were for patients with HGG only, 12 (20%) were open to patients with other gliomas, 4 (7%) were open to patients with other CNS tumors, and 1 (2%) to patients with other systemic tumors. Forty (68%) had a drug-only intervention with the remainder studying other types of interventions. Twenty-four (41%) had a start date between May 2009 and December 2014, within 5 years of bevacizumab FDA approval. Nineteen (32%) had a start date between January 2015 and December 2019, with the remaining 16 (27%) starting in 2020 or later.

## Discussion

### Barriers to Glioma Trial Accrual are Numerous and Delay Development of Novel Therapeutics

Despite the lack of proven therapies for recurrent GBM and other HGG, only an estimated 8%–11% of patients with GBM participate in clinical trials.^12^ Barriers to trial participation include patient, physician, and organization-related factors.^18^ Patients may lack an understanding of clinical trials, carry misperceptions about participation, or may be unable to participate due to limited access. Physicians may not be familiar with or lack incentives for seeking available studies. Organizations may lack necessary infrastructure. Increasingly narrow definitions of tumor types based on molecular characteristics may limit the availability of studies for rare tumor types. Additionally, there is a lack of diversity in brain tumor clinical trials with limited participation from minorities.^19^ These issues collectively increase time needed for trials to conclude and contribute to delays in development of novel therapeutics.^18^ Fewer barriers to enrollment may increase participation, facilitate timely trial completion, and address lack of diversity in clinical trial enrollment by improving the likelihood of minority accrual.

Excessively strict inclusion and exclusion criteria represent a major potential barrier to trial accrual. Maximizing opportunities for patients with recurrent HGG to participate in studies will expedite accrual and permit promising agents to move towards larger phase studies more quickly. This is particularly the case for early phase studies conducted to establish the safety and tolerability of pharmaceutical agents. For example, a limited course of bevacizumab is frequently used in clinical practice for treatment of radiation necrosis or symptomatic cerebral edema related to pseudoprogression.^20^ However, our data reveals that patients receiving any prior bevacizumab are frequently excluded from early-phase clinical trials.

### A Case for Unified Antiangiogenic Washout Rules

A blanket exclusion of patients with any prior bevacizumab use for recurrent glioma trials is difficult to justify. Such practice should be reserved for specific situations, such as the use of bevacizumab as a control arm or as part of the experimental intervention. Since bevacizumab is associated with wound-healing complications, exclusion of prior antiangiogenic administration for studies that involve a surgical resection or biopsy is reasonable.^21^ However, even in these circumstances, a washout period for bevacizumab may be preferred since normal wound healing 5–6 weeks following bevacizumab cessation has been reported despite effective circulating VEGF inactivation and a 4-week washout has been recommended prior to craniotomy.^22,23^ Despite this, 51 (17%) of the trials reviewed in this study precluded any prior bevacizumab or other antiangiogenic use without consideration for duration and intent of treatment. Thirty (59%) of these studies were early phase 1, phase 1, or phase 1/2 studies with 23 of them describing safety and tolerability as their primary objective (39%). Forty-one (69%) of these studies had a drug-only intervention with no requirement for a surgical procedure. For early-phase trials with a safety and tolerability endpoint or with no planned surgical intervention or surgical sample collection, exclusion of patients with any prior antiangiogenic use should be avoided. Prior therapy with a limited course of bevacizumab (or similar agents) should be permissible. Such an approach was utilized in 7 studies included in our analysis, but the specific implementations were varied. As an example, one study allowed up to 5 doses of bevacizumab if the agent was used for the management of radiation necrosis while 2 allowed less than 4 doses to be administered if the agent was used for cerebral edema only.

Requirement of a washout period for patients previously treated with bevacizumab is reasonable given previously reported concerns for rebound edema following abrupt bevacizumab discontinuation.^14^ Preclinical studies with spontaneous RIP-Tag2 tumors and implanted Lewis lung carcinomas in mice have been used to demonstrate vascular regrowth following reversal of anti-VEGF therapy, which represents a potential mechanism for rebound edema and accelerated tumor growth upon angiogenic discontinuation.^24^ However, in our analysis, antiangiogenic washout periods were widely varied, ranging from 2 to 24 weeks. The most common antiangiogenic washout period was 4 weeks, which was used in 35 studies (12% of the trials reviewed). In studies that did not list a rule specifically for bevacizumab or other antiangiogenics, general systemic therapy washout periods were also highly varied, ranging from 1–6 weeks or 3–5 half-lives of the prior therapy. The most common general systemic therapy washout was also 4 weeks, used in 34 studies (11% of the trials reviewed). In the case of bevacizumab, half-life is estimated at 20 days with 3 half-lives equaling approximately 9 weeks and 5 half-lives at approximately 14 weeks.^25^ Twenty-six (9% of the trials reviewed) required a specific antiangiogenic or other systemic therapy washout period that was longer than 4 weeks.

The heterogeneity of antiangiogenic washout rules in glioma trials is striking. There is little consistency in the rules utilized in various trials with designs ranging from blanket exclusion of patients who had any prior antiangiogenic exposure to studies open only for bevacizumab-refractory disease with no specified washout period. This heterogeneity applies to clinical trials utilizing agents with antiangiogenic activity besides bevacizumab. As an example, NCT01931098 utilized pazopanib and topotecan, permitting enrollment of patients with prior bevacizumab use if the most recent dose was administered at least 3 weeks prior to participation.^26^ On the other hand, NCT01817751 utilized sorafenib and excluded patients with any prior bevacizumab use.^27^ The impact of this heterogeneity on clinical trial outcomes is unknown. This heterogeneity is at least in part due to lack of large prospective studies evaluating the issue of bevacizumab discontinuation in patients with HGG. Optimal washout periods are unknown with no high-level data from large group prospective clinical trials to guide decision making. As such, washout rules utilized in clinical trial design remain arbitrary, likely reflective of individual investigator practice and preferences rather than scientific evidence. Nevertheless, a closer scrutiny of available retrospective and prospective data provides insights and guidance for future trial designs.

### Bevacizumab Discontinuation is Not Always Associated With Rebound Edema or Accelerated Progression

First, rebound tumor growth after bevacizumab discontinuation is relatively infrequent in patients with glioma, especially if bevacizumab is discontinued for reasons other than disease progression. For example, a retrospective review of 53 patients with recurrent HGG revealed that only 11 patients (21%) had rebound progression of disease after bevacizumab discontinuation.^14^ The mean interval between bevacizumab discontinuation and rebound disease progression was 6.1 weeks (median 6 weeks, range 3–10 weeks).^14^ Another study evaluated 7 patients with HGG who discontinued bevacizumab after an initial radiographic response but prior to tumor progression.^28^ Treatment was stopped due to toxicity in 4 cases and patient/physician choice in 3 cases. No patients experienced a rebound effect, and the median time to tumor recurrence after bevacizumab discontinuation was 4 months. In another review of 82 patients, 18 stopped treatment for reasons other than disease progression and no instances of rebound tumor growth were reported.^29^ Collectively, these data reveal that rapid tumor growth is relatively infrequent in patients with HGG when bevacizumab is discontinued for reasons other than disease progression.

Studies of other malignancies confirm that rebound disease growth is infrequent after bevacizumab discontinuation and may be disease-specific. For example, we have previously reported that rebound growth of vestibular schwannomas in neurofibromatosis type 2-related schwannomatosis occurs in a subset of patients within 6 months of bevacizumab discontinuation.^30^ However, other major malignancies do not seem to demonstrate any alterations in disease trajectory after bevacizumab discontinuation. In a pooled analysis, patterns of disease progression following bevacizumab discontinuation from 5 clinical trials representing 4205 patients with breast, colorectal, renal, and pancreatic cancer were described.^31^ No difference in median time from treatment discontinuation to progression or death and no evidence of accelerated disease was noted upon stopping antiangiogenic therapy due to adverse events. Similarly, there was no indication of increased mortality or decreased OS among patients where bevacizumab was stopped due to disease progression.^31^

Patients experiencing disease progression during bevacizumab therapy often continue to receive bevacizumab post-progression due to concerns about rebound tumor growth; however, the efficacy of this approach remains unclear. Continuation of bevacizumab following disease progression has been evaluated prospectively.^32^ As part of a phase 2 clinical trial, patients with recurrent GBM initially treated with bevacizumab were randomized to continue (*n* = 23) or cease bevacizumab (*n* = 25) with no difference in PFS or OS between the 2 groups. Median time to deterioration in overall quality of life for patients who continued bevacizumab was 1.15 (range.89–1.64) months, and 1.64 (range.85–2.04) months for those who ceased bevacizumab (HR = 1.25 for the continuation arm relative to the cessation arm, 95% CI: 0.70–2.24, *P* = .45) with steroid use similar between the 2 groups (median daily dexamethasone dose 4 mg in both arms).^32^In another clinical trial of adult patients with GBM, participants were randomized to lomustine plus bevacizumab (*n* = 61) or lomustine plus placebo (*n* = 62). Continued bevacizumab therapy was not associated with any survival benefit.^33^ Although the investigators did not specifically report on the incidence of rebound edema, there were no differences between treatment groups at the time of initiation and use of corticosteroids after randomization.^33^ These data suggest discontinuation of ongoing bevacizumab after disease progression does not seem to be associated with accelerated progression or rebound edema requiring disproportionate steroid use compared to patients who remain on the antiangiogenic therapy.

Collectively, these retrospective and prospective studies reveal a relatively limited risk of rebound edema, accelerated disease progression, and clinical decline after bevacizumab discontinuation in HGG. Therefore, these findings support the practice of enrolling patients with recurrent HGG in clinical trials after limited courses of prior antiangiogenic use. However, these studies provide less insight into the median time to development of rebound edema, and therefore offer insufficient guidance to select an optimal washout period after prior bevacizumab use.

### Pharmacokinetic, Pharmacodynamic, and Surgical Considerations for Antiangiogenic Washout

One way to define a washout period for bevacizumab or other antiangiogenics would be based on pharmacokinetic and pharmacodynamic properties. Bevacizumab is a monoclonal antibody with a long half-life estimated at 20 days, and thus an expectation for an extended duration of action.^25^ As an example, following intraocular administration, bevacizumab was associated with complete intravitreal VEGF blockade for a minimum of 4 weeks.^34^ In a study of colorectal cancer patients undergoing surgery, bevacizumab was found to be active and block circulating VEGF 6 weeks after cessation.^22^ Although this may provide some justification for prolonged bevacizumab washout periods, measurement of plasma VEGF may be insufficient in this regard. Clinical studies are conflicted in terms of whether or not plasma VEGF increases or decreases after bevacizumab therapy.^35,36^ Moreover, plasma VEGF levels are not associated with radiographic response.^36^ Surgical trials directly measuring glioma VEGF at various timepoints after bevacizumab withdrawal would be revealing, but very challenging to perform. Furthermore, the substantial inter-patient variability in both blood bevacizumab concentrations post-dosing (a mean 4-fold difference between lowest and highest concentration) and half-life (range = 11–50 days) adds additional layers of complexity in interpretation.^37,38^

An alternative strategy is to review the consequences of bevacizumab withdrawal on surgical complications. Several studies have examined perioperative risks involving the surgical resection of liver metastases in patients with colorectal cancer following neoadjuvant bevacizumab therapy. First, a comparative clinical trial demonstrated that discontinuation of bevacizumab at least 6 weeks prior to surgery was not associated with increased perioperative morbidity despite persistent effective VEGF inactivation.^22^ Similarly, a retrospective study demonstrated that neoadjuvant bevacizumab (discontinued at a median of 58 days prior to surgery) also revealed no difference in surgical complication rates.^39^ Limited studies have reported a 1%–10% rate of wound-healing complications for patients who previously received bevacizumab undergoing craniotomy.^7,23,40,41^ As an example, in a study of 209 patients who underwent a second or third craniotomy for recurrent GBM, significantly more patients receiving preoperative bevacizumab developed healing complications (35%) than non-bevacizumab-treated patients (10.0%, *P* = .004).^40^ While the total duration of bevacizumab therapy did not influence risk, there was a statistically nonsignificant trend toward increased risk of wound complications for patients who stopped bevacizumab for less than 28 days compared to those who had therapy cessation for at least 28 days.^40^ This led authors to recommend at least 4 weeks between bevacizumab cessation and surgical intervention whenever possible.^23,40^ Notably, the authors did not explore the impact of alternative durations (eg, 6 or 8 weeks) between the last dose of preoperative bevacizumab and craniotomy.

The practicality of antiangiogenic washout periods must also be considered for patients with HGG. The median PFS from next salvage therapy following bevacizumab discontinuation for disease progression was reported as 9 weeks in one study.^29^ In another study reviewing patterns of relapse and prognosis after bevacizumab failure, the median OS after progressive disease was 4.5 months.^42^ Among the 19 patients who received a salvage therapy following bevacizumab failure in the same study, the median PFS was 2 months and the median OS was 5.2 months.^42^ When bevacizumab was stopped for reasons other than tumor progression, the median PFS was reported as 4–6 months.^28,29^ In a pooled analysis of non-CNS tumors, median time from bevacizumab discontinuation due to adverse events to disease progression or death was 4 months.^31^ Based on available PFS and OS data, washout periods longer than 8 weeks are unlikely to be tolerated. Bevacizumab washout periods extending to 3 or 5 half-lives (approximately 9 and 14 weeks) are impractical.

### Guidance for Future Clinical Trial Designs

Determining the optimal washout period for patients with progressive disease on bevacizumab is challenging with limited data available to guide trial design recommendations and clinical decision making. We propose a 6-week bevacizumab washout (that is, approximately 2 half-lives) for patients with glioma enrolling in clinical trials, which is justified based on the earlier discussed (1) median time to rebound tumor progression following bevacizumab cessation previously reported as 6.1 weeks, (2) median time to clinical deterioration following bevacizumab discontinuation after disease progression from multiple studies indicating that washout periods longer than 8 weeks are unlikely to be tolerated, and (3) perioperative outcome data after neoadjuvant bevacizumab demonstrating relative safety of surgical intervention at least 4 weeks after bevacizumab discontinuation.^14^ A 6-week bevacizumab washout will avoid unnecessary delays in initiation of investigational therapeutics and is likely to be tolerated by most patients with recurrent glioma, while excluding the poorest candidates for clinical trial enrollment. This approach is also conservative enough to account for the impact of variabilities in bevacizumab dosing (7.5–15 mg/kg) and the significant inter-patient variability in bevacizumab pharmacokinetics. This is especially important for phase 1 trials where drug–drug interactions and novel drug pharmacokinetics are critical.

The impact of prior bevacizumab use on response assessment has also been a historical concern.^13,15^ In a survey of relapse patterns following bevacizumab use, it was concluded that contrast-enhanced MRI may not adequately assess disease status with a nonenhancing pattern of progression noted in some cases and associated with worse survival.^42^ However, in a different analysis of treatment with bevacizumab or bevacizumab plus irinotecan, most patients did not experience a change from baseline in radiographic characteristics of disease.^43^ Any impact of bevacizumab on response assessment is relatively less of a concern for phase 1 trials as these are primarily focused on safety and tolerability. Similarly, large phase 2 or 3 trials with OS endpoints should not be impacted. For phase 2 and 3 trials with imaging-based primary endpoints (response rate and progression-free survival), study design with an appropriate washout will enable broad participation of patients with prior bevacizumab use, leading to a trial which more accurately represents the patient population. Eventually, the impact of bevacizumab on efficacy can be addressed with planned subset analyses.

Shorter washout periods (<6 weeks) may be feasible in specific situations, especially in patients where bevacizumab was discontinued for reasons other than disease progression. Rebound/accelerated tumor growth after bevacizumab withdrawal is infrequent, and median corticosteroid needs do not seem to be impacted by drug discontinuation.^32,33^ The most common antiangiogenic-specific washout period specified in the clinical trials included in our analysis was 4 weeks, used in 35 studies (12% of the trials reviewed). Furthermore, a 2–3 week bevacizumab-specific washout was utilized in 5 of the studies in our analysis. However, based on information provided on ClinicalTrials.gov alone, one cannot ascertain the proportion of patients enrolled in these trials who actually received bevacizumab at these minimum timepoints prior to enrollment and the associated impact on outcomes.

Study designs permitting concurrent bevacizumab administration is another consideration, which mitigates concerns regarding rebound edema and associated clinical deterioration. Indeed, such a design was utilized in 5 clinical trials included in our analysis. Four of these were phase 1 studies with safety, tolerability, or pharmacokinetics-based primary endpoints while one was a phase 2 study with a PFS endpoint. Similar designs can be considered for clinical trials studying reirradiation or immunotherapy, which may mitigate issues such as radiation necrosis or pseudoprogression. In immunotherapy trials, utilization of antiangiogenics rather than steroids for management of tumor or treatment-related edema can be desirable to minimize immunosuppression related to corticosteroid use. Clinically stable patients with disease progression despite bevacizumab use could be considered for continuation of antiangiogenic therapy while participating in a study with an experimental agent. However, this approach would require consideration of potential drug–drug interactions, may add difficulty to adverse event assessment related to novel therapeutics, and would potentially require larger studies to permit result stratification based on concurrent use of antiangiogenics. One way to address this issue would be to require concurrent bevacizumab use, which would improve feasibility and limit the need for large sample sizes. Study designs specifically for patients with bevacizumab-refractory disease are another consideration, which was an approach implemented in 5 trials included in our analysis.

### Limitations and Future Directions

Our study has several limitations. First, the data presented is a snapshot of studies acquired on the search date and does not represent changes to participant eligibility rules that may have occurred with subsequent protocol amendments. Second, the analysis is based on study data as presented on ClinicalTrials.gov, which contains summary-level information rather than full study protocol details. Third, our search is limited to ClinicalTrials.gov and omits any studies that may not have a listing on this platform. Lastly, since our analysis of the listed trials does not include individual patient-level data as to how antiangiogenic rules were reflected in the real-world experience of study participants, it is difficult to strongly recommend rules for future study design on the basis of incomplete information. Nevertheless, this is a survey of a large number of clinical trials enrolling patients with HGG and likely captures antiangiogenic-related eligibility trends within the field of neuro-oncology during our search period. In addition, we attempted to consider reported clinical experience with bevacizumab discontinuation in the setting of glioma and other cancers, pharmacokinetics of bevacizumab, and reported neurosurgical experience related to post-operative complications to frame our recommendations for future study design. We view our presented work as a starting point that can be refined and prospectively validated.

Given universally poor outcomes for patients with HGG, there is a desperate need to bring new treatments into the clinic. These deadly tumors will be ultimately overcome by novel therapeutics and great science. However, equally important is diligent clinical trial design that puts the needs of patients first, encourages participation, and takes down barriers rather than making it harder for patients to join. Our analysis demonstrates that antiangiogenic-based eligibility rules used in HGG clinical trials are inconsistent and heterogeneous. Available clinical data does indicate bevacizumab discontinuation for reasons other than disease progression is not associated with rebound edema, accelerated tumor growth, or shortened survival. Bevacizumab discontinuation for disease progression can result in rebound edema, but this is not universal. A 6-week washout is likely to be tolerated by most patients, represents an established safe window for craniotomy, and permits timely introduction of experimental therapeutics. Implementation of practical washout periods and avoiding the practice of excluding patients with any prior antiangiogenic use will increase enrollment and expedite study completion.

## Supplementary Material

vdae039_suppl_Supplementary_Tables_1

## Data Availability

Study data will be made available upon reasonable request.
