# Supplementary material for: Antiangiogenic exclusion rules in glioma trials: Historical perspectives and guidance for future trial design
Source: Neurooncol Adv. 2024 Mar 15;6(1):vdae039. doi: 10.1093/noajnl/vdae039 (PMC11003534; doi:10.1093/noajnl/vdae039)
Supplement: vdae039_suppl_Supplementary_Tables_1 [file vdae039_suppl_supplementary_tables_1.docx]

**Supplementary Table 1: Recurrent Glioma Clinical Trial Target Accruals and Funding Sources**

| **Characteristic** | **n (%)** |
| --- | --- |
| Total studies reviewed | 297 |
| Target enrollment  0 – 24  25 – 49  50 – 99  100 – 199  200 – 200+ | 109 (37%)  92 (31%)  50 (17%)  30 (10%)  16 (5%) |
| Funding source  Industry only  NIH only  Other only*  Combination with industry  Combination non-industry** | 86 (29%)  23 (8%)  71 (24%)  79 (27%)  38 (13%) |

* Other indicates studies funded by institutions other than industry and NIH.

** Includes studies funded by NIH, other U.S. Federal Government funding, or other non-industry sources such as academic institutions.
